# The Biological Roles of microRNAs in *Drosophila* Development

**DOI:** 10.3390/insects15070491

**Published:** 2024-06-30

**Authors:** Daegyu Jang, Chae Jeong Kim, Bo Hyun Shin, Do-Hwan Lim

**Affiliations:** School of Systems Biomedical Science, Soongsil University, Seoul 06978, Republic of Korea; sjj609349@gmail.com (D.J.); jinrourin313@naver.com (C.J.K.); breadott@naver.com (B.H.S.)

**Keywords:** microRNA, *Drosophila*, development, growth, apoptosis, eye, wing, nervous system

## Abstract

**Simple Summary:**

*Drosophila* (or the fruit fly) is widely used to study developmental processes associated with gene regulation. Insect development, including that of *Drosophila*, is controlled by various gene regulation mechanisms. In particular, numerous studies on microRNAs (miRNAs) in *Drosophila* have highlighted their crucial roles in development. This review focuses on the function of miRNAs in various tissues and developmental processes in *Drosophila*, along with sequencing technologies for studying miRNA interactions with target genes.

**Abstract:**

*Drosophila* is a well-established insect model system for studying various physiological phenomena and developmental processes, with a focus on gene regulation. *Drosophila* development is controlled by programmed regulatory mechanisms specific to individual tissues. When key developmental processes are shared among various insects, the associated regulatory networks are believed to be conserved across insects. Thus, studies of developmental regulation in *Drosophila* have substantially contributed to our understanding of insect development. Over the past two decades, studies on microRNAs (miRNAs) in *Drosophila* have revealed their crucial regulatory roles in various developmental processes. This review focuses on the biological roles of miRNAs in specific tissues and processes associated with *Drosophila* development. Additionally, as a future direction, we discuss sequencing technologies that can analyze the interactions between miRNAs and their target genes, with the aim of enhancing miRNA studies in *Drosophila* development.

## 1. Introduction

Development is a delicate process controlled by a variety of external and internal factors. These factors ultimately influence the temporal and spatial regulation of genes involved in various developmental processes. Gene expression is regulated in two steps, namely transcription and post-transcription. Various transcription factors control the expression of numerous genes, orchestrating intrinsic cellular programs for proper development. Subsequently, after transcription, individual RNAs can be regulated by diverse mechanisms, including suppression mediated by small non-coding RNAs called microRNAs (miRNAs) [1]. 

*Drosophila* serves as an attractive insect model system for scientists studying developmental processes regulated by various gene regulatory mechanisms. This is because a combination of classical genetics with a variety of molecular, cellular, and biochemical techniques can be easily employed to study *Drosophila* development. The generation time of *Drosophila* from fertilized eggs to adult flies is approximately 10 days [2]. During this period, *Drosophila*, a holometabolous insect, undergoes four stages, namely embryonic, larval, pupal, and adult stages of development [2]. At each stage, individual cells differentiate into specific cell types and form distinct tissues under programmed signals. This review focuses on developmental processes regulated by miRNAs.

Given that gene regulation mechanisms are important in the development of insects, including *Drosophila*, it is not surprising that miRNAs controlling gene expression have emerged as central regulators of development. Since the discovery that *lin-4* plays a role in controlling the temporal development of *Caenorhabditis elegans* by negatively regulating lin-14 [3,4], efforts have been made to identify miRNAs generated from *Drosophila* using molecular biology and biochemistry techniques [5]. In the study, 16 miRNAs, including *miR-1*, *miR-2b*, and *miR-6*, were identified in *Drosophila* and S2 cells using a cloning method employing adapter ligation. The study also analyzed the expression patterns of these miRNAs at several developmental stages, including embryos, larvae, pupae, and adults. Some of the discovered miRNAs, such as *miR-13a* and *miR-13b-1*, were clustered. Through further efforts employing small RNA cloning, a non-redundant set of 62 miRNAs, including *miR-34*, *miR-92a*, and *miR-184*, was identified across various developmental stages, with the expression profiling of individual miRNAs [6]. In this study, the miR* sequences, which represent the opposite strand of the identified primary strand of miRNAs, were also cloned for some miRNAs (miR-10*, miR-13a*, and miR-281-2*), despite being relatively less abundant.

Subsequent studies employing computational approaches and high-throughput sequencing technologies led to the discovery of many novel miRNAs, including those with low expression levels [7,8]. According to the miRBase an miRNA database, 469 mature miRNAs have been identified in *Drosophila melanogaster* to date [9]. Despite the discovery of several hundred miRNAs in *Drosophila*, studies of their biological roles in developmental processes are limited. 

The knowledge of known gene regulatory roles of miRNAs in development should be comprehensively summarized and shared with researchers in this field. A previous insightful review explained the biological roles of some known miRNAs in various developmental processes [10]. Additionally, a more recent review focused on miRNAs involved in embryonic development and specific developmental aspects, such as muscle development and homeotic gene regulation [11]. In this review, with recent additional results, we summarize the basic miRNA pathways in *Drosophila* and the known biological roles of miRNAs in relatively well-explored tissues, such as the nervous system, wing, and eye, as well as in processes associated with development, such as growth and cell death. Rather than attempting to cover all related literature on development-associated miRNAs, we focus on these specific areas. Additionally, we briefly discuss recent sequencing techniques that can be used to enhance our understanding of the regulatory function of miRNAs in *Drosophila*. 

## 2. miRNA Biogenesis and Mechanism in *Drosophila*

**Figure 1:**
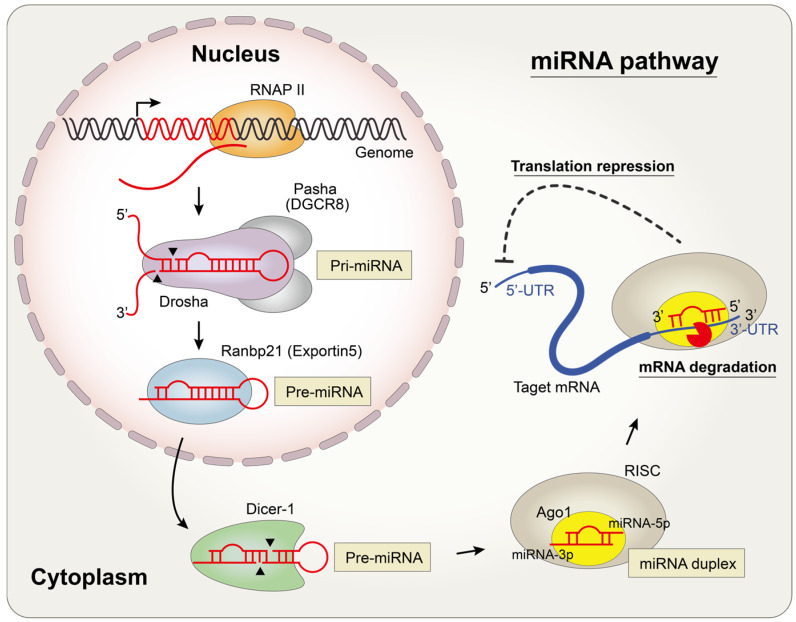
miRNA biogenesis and regulatory mechanism in *Drosophila*. Pri-miRNAs are transcribed by RNAPII in the nucleus and cleaved into pre-miRNAs by the microprocessor complex, which includes Drosha and Pasha. After pre-miRNAs are exported to the cytoplasm by Ranbp21, pre-miRNAs are further processed into miRNA duplex by Dicer-1. Finally, miRISC, containing a single-strand miRNA, suppresses the expression of specific genes by translation repression and/or mRNA degradation.

The miRNA genes are encoded in the genome and transcribed by RNA polymerase II (Figure 1) [12]. As features of RNAs transcribed by RNA polymerase II, the resulting primary transcripts, known as primary miRNAs (pri-miRNAs), contain 5′ 7-methyl caps and 3′ poly-A tails [13]. These pri-miRNAs can be transcribed by several kilobases and often encompass multiple distinct miRNAs in clustered miRNA sets. A minor group of miRNAs is alternately transcribed by RNA polymerase III [14]. 

Pri-miRNAs, which contain stem–loop structures, undergo processing into precursor miRNAs (pre-miRNAs) of approximately 70 nucleotides (nt) in length. This process occurs via the endonucleolytic cleavage of the stem of the hairpin structure [15]. The cleavage process is catalyzed by the microprocessor complex comprising Drosha, a nuclear ribonuclease III (RNase III), and the Partner of Drosha (Pasha) in *Drosophila* (DGCR8 in humans) [16,17,18]. The cropping of pre-miRNAs from pri-miRNAs by Drosha occurs in a co-transcriptional manner [19]. However, a distinct type of miRNA known as mirtron, which bypasses the Drosha-mediated cleavage step, has been identified through high-throughput sequencing analysis in *Drosophila* [20,21]. Without Drosha processing, these mirtrons are processed into lariat mirtrons by spliceosomal machinery. Subsequently, they form structural mimics of the pre-miRNAs via a debranching process.

Cleaved pre-miRNAs exhibit high affinity for Ranbp21 (known as exportin 5 in humans) and are subsequently exported from the nucleus to the cytoplasm through the pre-miRNA–Ranbp21 interaction [22,23,24]. Depletion of Ranbp21 inhibits the export of pre-miRNAs, resulting in a reduction in mature miRNAs [22]. 

Pre-miRNAs are further processed into approximately 21 nt miRNA duplexes by the RNase III enzyme, Dicer (Dcr), in the cytoplasm [25,26,27]. In *Drosophila*, there are two Dcr proteins, Dcr-1 and Dcr-2, which play distinct roles [28]. Dcr-1 and Dcr-2 function in miRNA and siRNA pathways, respectively. Dcr recognizes the 2 nt 3′ overhang of pre-miRNAs using its PAZ domain and subsequently cleaves the stem of pre-miRNAs approximately 20 nt away [29,30]. This cleavage process yields an miRNA duplex. 

These miRNA duplexes facilitate mRNA repression through an effector complex known as the miRNA-induced silencing complex (miRISC) [31]. The core protein within the miRISC is the Ago protein. In *Drosophila*, similar to Dcr, Ago1 and Ago2 are closely associated with miRNA and siRNA pathways, respectively [32,33]. Upon incorporation of the miRNA duplexes into Ago1, the two strands of the miRNA duplexes are dissociated, and eventually, one strand of the miRNA duplex, serving as the guide, is retained within the miRISC [34,35]. The strand of the miRNA incorporated into Ago1 is named with a −5p or −3p suffix, depending on whether it is generated from the 5′ or 3′ arm of the stem region in the pre-miRNA [36].

miRNAs generally bind to the 3′-untranslated region (3′-UTR) of target mRNAs in a sequence-specific manner, ultimately resulting in the inhibition of gene expression by suppressing translation and/or promoting mRNA decay [1]. 

## 3. Regulation of miRNA Expression by Hormones in *Drosophila*

*Drosophila* hormones regulate development. Among these, two essential hormones, ecdysteroids and juvenile hormones (JHs), are closely associated with the control of developmental timing and processes. 

JH is produced by the corpus allatum of the ring gland [37]. Initially, JHs bind to methoprene-tolerant (Met) or germ cell-expressing JH intracellular receptors [38]. Met forms a complex with Taiman (Tai) and heat shock protein 83 (Hsp83) [39,40], and subsequently, Nucleoporin 358 kD (Nup358) facilitates the translocation of this complex into the nucleus [41]. This JH-Met complex binds to the JH response region and activates the expression of JH-responsive genes such as zinc-finger TF Krüppel-homolog 1 (*Kr-h1*) [37].

Ecdysone is released from the prothoracic glands of the ring gland [42]. Canonical ecdysone signaling is initiated by the binding of the active ecdysone metabolite, 20-hydroxyecdysone (20E), to a heterodimeric receptor comprising an ecdysone receptor (EcR) and ultraspiracle (USP) [43,44,45]. This complex directly activates the expression of a small set of primary response genes, such as broad (*br*), ecdysone-induced protein 74EF (*E74*), and ecdysone-induced protein 75EF (*E75*) [46,47,48]. These early ecdysone-responsive transcription factors then regulate a large set of secondary response genes encompassing both protein-coding and non-coding genes [49,50].

These hormone-signaling pathways regulate the expression of miRNAs that serve as central gene regulators, in addition to the regulating protein-coding genes. The dynamic expression of miRNAs, influenced by hormones, can lead to drastic alterations in the expression of various genes, such as those observed during metamorphosis [50,51], or fine-tune the expression of individual genes, as seen in differentiation processes.

Some research groups have identified miRNAs that respond to the titration of each hormone and are associated with developmental stages highly influenced by specific hormone activities. In the early stages of miRNA discovery in *Drosophila*, Lorenzo et al. found that *miR-100*, *miR-125*, and *let-7*, which are upregulated during the larval-to-pupal transition, also show increased expression in response to ecdysone hormones in S2 cells, as determined by northern blotting [52]. These three miRNAs (*let-7-Complex*, *let-7-C*) are co-transcribed and involved in normal adult behaviors through the regulation of remodeling of the abdominal neuromusculature [53]. In contrast, *miR-34* expression was lower in 20E-treated S2 cells compared with that in untreated control cells. A study using cross-linking immunoprecipitation with deep sequencing (CLIP-seq) revealed that 30 Ago1-associated miRNAs (including *miR-252-5p*, *miR-276a-3p/5p*, and *miR-279-3p*) were upregulated, while 42 Ago1-associated miRNAs (including *miR-317-3p* and *miR-8-5p*) were downregulated following 20E treatment in S2 cells [50]. In another study using small RNA sequencing, 43 upregulated miRNAs (including *miR-278-3p/5p*, *miR-306-5p*, and *miR-13a-3p*) and 35 downregulated miRNAs (including *miR-970-5p*, *miR-979-3p*, and *miR-998-3p*) were identified in S2 cells treated with 20-hydroxyecdysone [54]. 

In addition to ecdysone-responsive miRNAs, Lorenzo et al. identified *miR-34* as a JH-responsive miRNA in S2 cells [52]. Other studies found that the expression of *miR-927*, *let-7*, *miR-100*, and *miR-125* is repressed in S2 cells by methoprene, a JH analog [55,56,57]. However, relatively few studies have been conducted on JH-responsive miRNAs compared with those on ecdysone-responsive miRNAs. 

## 4. Biological Roles of miRNAs in *Drosophila* Development

A set of miRNAs is regulated by programmed signals, which subsequently affect various target genes involved in developmental processes. Here, we discuss the developmental processes in which the roles of miRNAs have been relatively well explored and review the functions of these miRNAs (Table 1).

### 4.1. Developmental Growth and Apoptosis

Proper regulation of growth and programmed cell death, including apoptosis, at each developmental stage is crucial for the developmental process. To date, many research studies have unveiled several miRNAs associated with these biological processes. 

#### 4.1.1. miRNAs Involved in Developmental Growth

According to previous observations, miRNAs upregulated by the ecdysone signaling pathway are typically associated with negative regulation of growth, whereas downregulated miRNAs are likely involved in positive regulation of growth.

Ecdysone-induced miRNA, *miR-252*, is expressed and functions in the fat body during the larval stage [50,58]. A lack of *miR-252* leads to increased body weight in adult flies, while overexpression of *miR-252* in the fat body results in a significant reduction in developmental growth, accompanied by a lower cell number and smaller cell size. Growth alteration upon the change in *miR-252* expression is mediated by targeting mushroom bodies tiny (*mbt*), which encodes a p21-activated kinase. Additionally, *miR-252* controls the cell cycle by regulating cyclins A and B by suppressing Abelson interacting protein (*Abi*) [50]. This also affects the developmental growth of *Drosophila*. 

**Figure 2:**
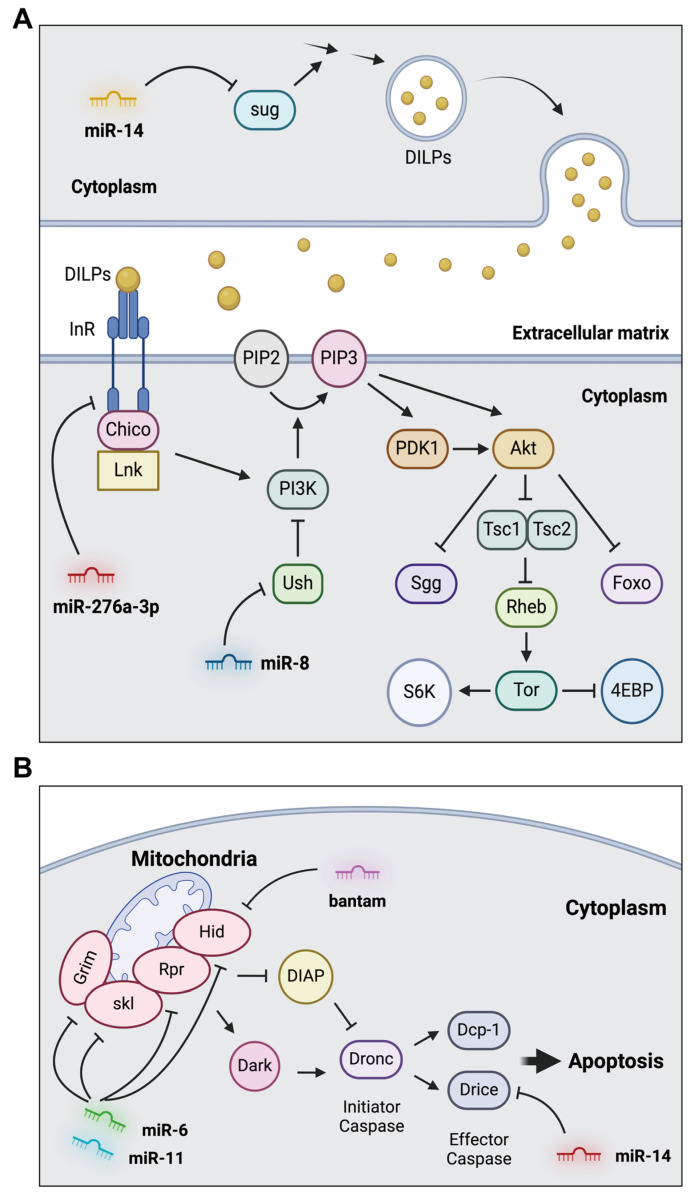
The roles of miRNAs involved in the developmental growth and apoptosis of *Drosophila*. (**A**) *miR-276a*, *miR-8*, and *miR-14* mediate the regulation of the insulin/insulin-like growth factor signaling pathway. (**B**) *Bantam*, *miR-14*, *miR-6*, and *miR-11* control the apoptosis signaling pathway.

In addition to cell cycle control, the insulin/insulin-like growth factor signaling (IIS) pathway plays a central role in developmental growth in *Drosophila*. The IIS pathway is activated by the binding of *Drosophila* insulin-like peptides (dILPs) to Insulin-like receptors (InRs) (Figure 2A) [83]. In *Drosophila*, eight dILPs (dILPs 1-8) have been identified and are expressed in insulin-producing cells in the brain, visceral muscles of the gut, and fat body. Through the binding of dILPs to InR in the target tissues, docking proteins Chico (IRS fly ortholog) and Lnk (fly ortholog of SH2B adaptor proteins) were recruited [84]. Chico phosphorylates phosphoinositide-3 kinase (PI3K), transferring the signaling to phosphoinositide-dependent kinase 1 (PDK1) and Akt kinase (Akt1). Subsequently, this signaling pathway expands to the forkhead box—subgroup O (FOXO), shaggy (sgg), or Tsc1/Tsc2 complex [84]. This signaling cascade significantly impacts the developmental growth of *Drosophila*.

*miR-276a*, another ecdysone-induced miRNA, is also expressed in larval fat bodies [60]. When *miR-276a* is upregulated in the fat body, the total mass of the larval fat body is dramatically reduced, resulting in developmental growth defects. This defect is associated with the inactivation of insulin signaling by *miR-276a-3p*-mediated suppression of InR (Figure 2A). In contrast, *miR-8*, which is downregulated by ecdysone signaling, is implicated in the inhibition of the IIS pathway [62]. Deletion of *miR-8* in flies results in a small body size with inactive IIS in the fat body. This phenotype is linked to the PI3K inhibitor-encoding u-shaped (*ush*), which is a target of *miR-8* (Figure 2A). Moreover, *miR-14* is downregulated by ecdysone signaling and controls the production of dILPs in insulin-producing neurosecretory cells [64,85]. Mutants lacking *miR-14* show decreased dILP3 and dILP5 production. Regulation by *miR-14* is achieved by targeting sugarbabe (*sug*) (Figure 2A). 

#### 4.1.2. miRNAs Involved in Programmed Cell Death

Programmed cell death, including apoptosis, is closely associated with *Drosophila* development. In *Drosophila*, various apoptotic stimuli induce the transcriptional expression of reaper-family genes, including reaper (*rpr*), head involution defective (*hid*), and *grim* (Figure 2B) [86]. These proteins can act as antagonizing inhibitors of *Drosophila* IAP-1 (Diap1), which functions as an E3-ubiquitin ligase, promoting the degradation of the initiator caspase, death regulator Nedd2-like caspase (Dronc) [86]. Dronc is activated by the Dark apoptosome and subsequently transduces apoptotic signals by cleaving and activating the effector caspases, namely Death related ICE-like caspase (Drice) and death caspase-1 (Dcp-1) [87]. 

*Bantam* miRNA was identified as an miRNA that promotes cell proliferation [66]. Analysis using an miRNA sensor revealed that *Bantam* activity was correlated with cell proliferation in the developing larval brain and wing discs. *Bantam* miRNA negatively regulates the expression of *hid*, resulting in the inhibition of hid-mediated apoptosis in *Drosophila* (Figure 2B) [66].

Additionally, *miR-14* suppresses *Drosophila* cell death [65]. Reduced *miR-14* activity leads to severe eye defects induced by Rpr. Conversely, eye-specific expression of *miR-14* alleviates retinal cell death induced by Rpr, Hid, Grim, and Dronc [65]. The anti-apoptotic effect of *miR-14* is likely achieved by regulating the levels of Drice (Figure 2B).

Furthermore, *miR-6* and *miR-11* are involved in the development of the embryonic central nervous system (CNS) through the regulation of the pro-apoptotic genes *rpr*, *hid*, *grim*, and sickle (*skl*) (Figure 2B) [68]. Recently, screening for anti-apoptotic miRNAs was performed in a *Drosophila* eye model induced by the misexpression of pro-apoptotic genes such as *hid* and *grim* [88]. From the screening, 16 miRNAs, including *miR-7* and *miR-125*, were identified as apoptosis suppressors in both the GMR-hid and GMR-grim genetic backgrounds.

### 4.2. miRNAs in the Development of the Nervous System

Studies have revealed that miRNAs play important roles in the development of the nervous system in *Drosophila*. These miRNAs influence various developmental processes in the nervous system, including the growth, differentiation, structural formation, and maintenance of nerve cells, by regulating specific target genes in distinct cell types. 

#### 4.2.1. *miR-92a/b*, *Bantam*, and *miR-124* in Neuroblasts (NBs)

The *Drosophila* nervous system is derived from neural stem cells known as NBs [89]. These NBs undergo asymmetric cell division to generate different neuronal subtypes. The self-renewal, differentiation, and proliferation of NBs are regulated by intrinsic gene regulatory programs, including miRNAs.

Both *miR-92a* and *miR-92b* are highly expressed in the NBs of the *Drosophila* larval brain [72]. *miR-92a* is located in the first intron of Jing interacting gene regulatory 1 (*jigr1*), while *miR-92b* is in the 3′-UTR downstream of the *jigr1* coding region. Consequently, these two miRNAs are co-expressed with *jigr1*. In *miR-92a/b* knockout mutant flies, the number of NBs is significantly reduced. However, this reduction is not linked to an increase in apoptosis or defects in asymmetric cell division [72]. Instead, the loss of *miR-92a/b* leads to premature differentiation and reduced proliferation of neuroblasts. Due to these defects, *miR-92a/b* mutant flies exhibit a smaller ventral nerve cord and brain compared to control flies. *MiR-92a/b* negatively regulates the host gene, *jigr1*. Similarly, overexpression of *jirg1* in NBs also results in a reduction in the brain size of adult flies due to the premature differentiation of NBs [72]. 

*Bantam* has been detected in the neuronal progenitor cells of the *Drosophila* larval brain [67] and is implicated in the control of NB proliferation. *Bantam* deletion mutants exhibit a smaller CNS and a decrease in both type I (producing ganglion mother cells) and type II (producing intermediate neural progenitors) NBs in the larval central brain. These phenotypes are associated with reduced expression of brain tumor (*brat*) and prospero (*pros*) genes, which are targeted by *bantam*. 

*miR-124* is expressed in proliferating neuronal progenitors and substantially contributes to the development of the CNS by targeting anachronism (*ana*), which encodes a secreted inhibitor of NB proliferation [73]. Studies using targeted *miR-124* knockout mutants have demonstrated that *miR-124* is crucial for neuronal progenitor cell proliferation. 

#### 4.2.2. *Let-7* and *miR-iab8* in the Mushroom Body (MB)

**Figure 3:**
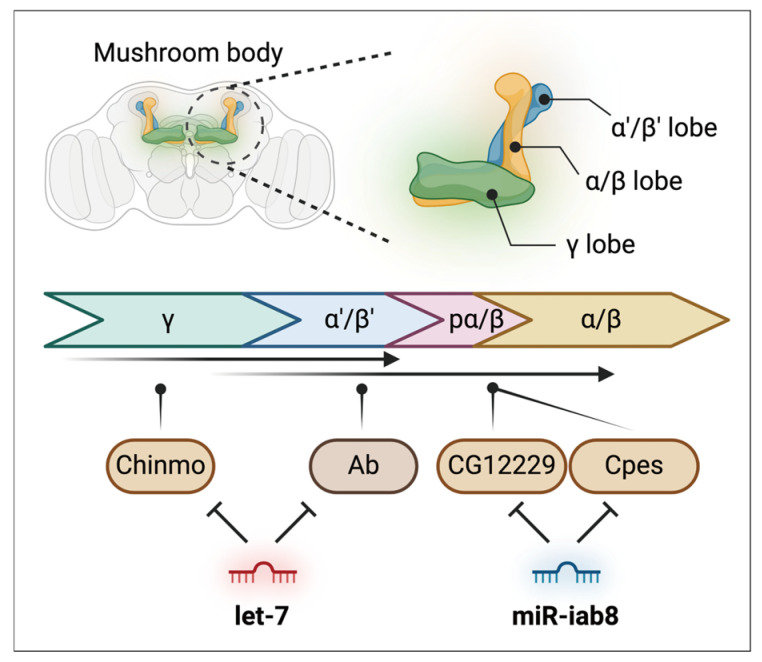
Involvement of miRNAs in the mushroom body (MB) development of *Drosophila*. *Let-7* and *miR-iab8* regulate the generation of MB neurons by controlling the transition of MB subtypes.

The MB is a central tissue for olfactory learning and memory, comprising four neuronal cell subtypes (γ, α′/β′, pioneer α/β, and α/β) that are generated in a specific order during *Drosophila* development (Figure 3) [90,91]. 

Several miRNAs are involved in the formation of MBs. *Let-7*, an ecdysone-induced miRNA, plays an important role in regulating the timing of neuronal development in the MB [69]. By targeting Chronologically inappropriate morphogenesis (*chinmo*), which encodes a putative BTB-zinc finger transcription factor, *let-7* controls the developmental timing of neurons in the MB (Figure 3). The loss of *let-7* leads to a delay in the transition of MB subtypes (γ → α′/β′ → pioneer α/β → α/β) by inhibiting *chinmo*, which drives the sequential generation of MB neurons. 

In addition, *let-7* regulates the timing of the transition from α′/β′ to α/β neuronal identities in the mushroom body by targeting another transcription factor, namely Abrupt (*Ab*) (Figure 3) [70]. The *let-7-Ab* regulatory axis influences the expression of cell adhesion molecule Fasciclin 2 (*Fas2*) in developing neurons. Consequently, the absence of *let-7* results in learning defects due to the abnormal morphology of the MB. 

Another crucial miRNA, *miR-iab8*, is involved in the formation of MB neurons [71]. Depletion of *miR-iab8* using an miRNA sponge (SP) in the CNS and MB leads to reduced intermediate-term memory. Additionally, *miR-iab8-SP*-mediated inhibition causes a decrease in the number and distribution of α/β MB neurons [71]. Researchers have identified *CG12229*, which encodes pyruvate kinase and Ceramide phosphoethanolamine synthase (*Cpes*), as a potential target associated with these defects (Figure 3). Knockdown of these genes resulted in improved memory performance, contrary to the results observed with the depletion of *miR-iab8* [71].

#### 4.2.3. *miR-34*, *miR-124*, and *miR-276a/b* in Synaptogenesis and Dendritic Formation

*miR-34* plays a crucial role in synaptogenesis in *Drosophila* [75]. Using RNAi, *miR-34* was initially identified as an miRNA that promotes neuromuscular junction (NMJ) growth. In *miR-34* mutants, defects in NMJ synapse formation have been observed, including a reduction in the expansion of motor axon terminal arbors and a decrease in the number of presynaptic boutons. Notably, *miR-34* is involved in NMJ formation via distinct mechanisms in presynaptic and postsynaptic neurons [75]. In the presynaptic compartment, *miR-34* inhibits junctional adhesion receptor Neurexin IV (Nrx-IV), thereby affecting active zone formation. In contrast, in the postsynaptic compartment, *miR-34* suppresses the expression of membrane cytoskeletal effector protein Hu li tai shao (Hts), thereby controlling the initiation of bouton formation [75]. 

Furthermore, *miR-124* is expressed in the CNS of *Drosophila*, particularly in proliferating neuronal progenitors and differentiating postmitotic neurons [73,74]. Studies have confirmed its biological significance in CNS development. Deletion of *miR-124* leads to increased dendritic arborization, impaired larval locomotion, and abnormal synaptic transmission at NMJs [74]. *miR-124* coordinately suppresses five positive components (saxophone [*sax*], thickveins [*tkv*], wishful thinking [*wit*], Mothers against dpp [*Mad*], and Medea [*Med*]) of the retrograde BMP signaling pathway in neurons. 

*miR-276* is associated with conserved RNA-binding protein fragile X mental retardation protein (FMRP), which is involved in dendritic arbor morphogenesis [61]. The activity of *miR-276a/b* is linked to the dendritic field coverage of *Drosophila* larval class IV da (C4da) sensory neurons by suppressing the target gene, nejire (*nej*). Nej functions as a transcriptional co-activator, regulating the expression of genes related to cytoskeletal dynamics, which are essential for dendritic formation. The *miR-276–nej* interaction is crucial for proper dendritic branching in neurons that receive signals from other neurons.

### 4.3. miRNAs in Wing Development

*Drosophila* wing development is regulated by multiple signaling pathways, such as the Hedgehog (Hh), Notch, and Wingless (Wg) pathways. Through these signaling pathways, cell proliferation, boundary formation, patterning, and morphogenesis in wing discs are regulated. Here, we discuss the regulatory roles of specific miRNAs in the signaling pathways associated with wing development.

#### 4.3.1. *miR-960* and *miR-5* in the Hh Signaling Pathway of Wing Development

**Figure 4:**
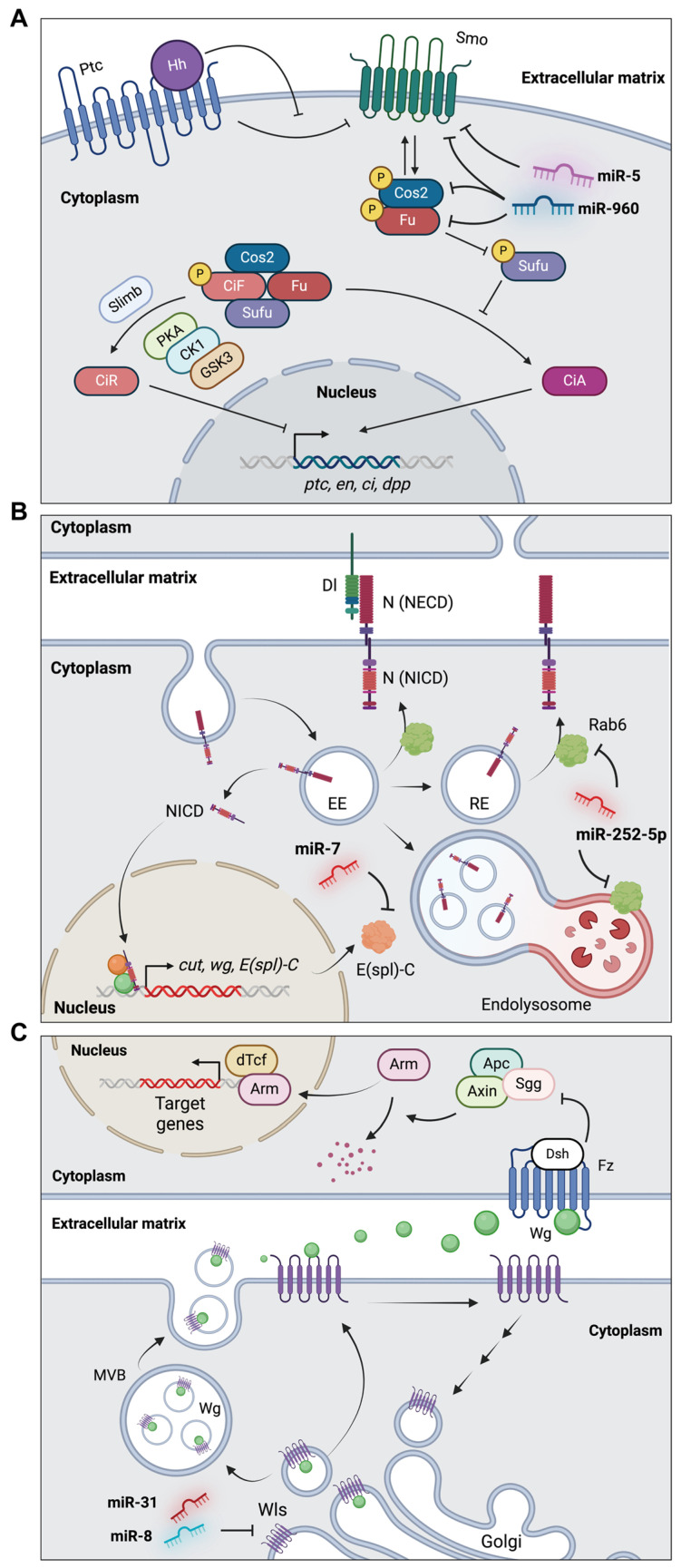
The signaling pathways regulated by miRNAs in wing development. (**A**) *miR-5* and *miR-960* control the Hh signaling pathway by targeting specific signaling component genes. (**B**) *miR-7* and *miR-252* regulate the Notch signaling pathway. (**C**) *miR-31a/b* and *miR-8* are involved in the Wg signaling pathway.

Distinct regulation occurs in the anterior and posterior compartments of the wing disc during wing imaginal disc development. Notably, the expression of Hh is restricted to the posterior compartment and diffuses to influence the adjacent anterior cells [92,93]. In the absence of the Hh ligand, Patched (Ptc) inhibits Smoothened (Smo), which results in the suppression of the kinase activity of the Hh complex (PKA, CK1, and GSK3) (Figure 4A) [94,95]. This suppression leads to the phosphorylation of the supernumerary limbs (Slimb), which facilitates the generation of the truncated repressor form of Cubitus interruptus (Ci) via proteasomal proteolysis. The repressor form of Ci (CiR) inhibits the expression of Hh target genes in the nucleus. In contrast, when the Hh ligand binds to Ptc, the Ptc-mediated inhibition of Smo is relieved. [94,95]. Subsequent phosphorylation by PKA and CKI leads to the formation of the Costal-2 (Cos2)-Fused (Fu) complex, leading to inhibition of the Suppressor of Fused (Sufu) complex, which suppresses Ci activation [94,95]. Consequently, the full-length active form of Ci activates Hh-dependent genes in the nucleus.

One group studied the regulation of Hh signaling by *miR-5* in *Drosophila* wings [77]. *miR-5* was identified as a negative regulator of the Hh signaling pathway by suppressing *smo*. Overexpression of *miR-5* in the wing pouch leads to a reduced intervein region between longitudinal veins 3 and 4 (L3 and L4), which is associated with Hh signaling. Furthermore, *miR-5* suppresses the expression of Hh signaling target genes such as engrailed (*en*), *ptc*, *ci*, and decapentaplegic (*dpp*). This suppression was achieved by direct targeting of *smo* (Figure 4A) in [77]. In addition, *miR-5* appears to inhibit the Wg signaling pathway by regulating other genes and is not associated with the Notch or Dpp signaling pathways, as determined by the unchanged expression levels of Cut (ct) and Spalt major (salm), respectively [77].

Another study revealed that *miR-960* suppresses Hh signaling by directly inhibiting Smo, Cos2, and Fu (Figure 4A) [76]. When the *miR-960* cluster (*miR-960C*), comprising *miR-959*, *miR-960*, *miR-961*, and *miR-962*, is overexpressed in the wing pouch, the L3-L4 intervein region is significantly reduced. Moreover, the overexpression of *miR-960C* in the dorsal compartment of the wing pouch results in a marked decrease in the expression of Ptc, Col, and Ci [76].

#### 4.3.2. *miR-7* and *miR-252* in the Notch Signaling Pathway of Wing Development

As another well-known crucial signaling pathway, the Notch signaling pathway is primarily involved in cell proliferation and differentiation throughout *Drosophila* development, particularly in the formation of the dorsal/ventral boundaries and wing-margin patterning within the wing imaginal disc [96]. In the canonical Notch signaling pathway, the interaction of the Notch receptor with membrane-bound ligands, such as Delta (Dl) and Serrate (Ser), between neighboring cells triggers the activation of Notch signaling (Figure 4B) [97,98]. This leads to two proteolytic cleavages, resulting in the release of the Notch intracellular domain (NICD). The NICD then translocates to the nucleus to activate the expression of target genes, including *wg* and *ct* [97,98]. 

*miR-7* is expressed in wing imaginal discs [99]. Depletion of *miR-7* results in reduced wing size, whereas overexpression in the wing pouch induces wing-margin notching, which is indicative of diminished Notch signaling. Consistent with this finding, the expression of *ct* is increased in *miR-7*-overexpressing wing discs. This effect may be related to the regulation of Enhancer of split complex (*E(spl)-C*) genes by *miR-7*, as determined in previous studies (Figure 4B) [78,79].

Another miRNA associated with the Notch signaling pathway, *miR-252-5p*, regulates Notch signaling by targeting *Rab6*, a member of the Rab family of small GTPases (Figure 4B) [59]. Overexpression of *miR-252* in the posterior compartment of the wing disc leads to wing defects accompanied by notching of the wing blade and decreased expression of *ct*. Notably, *miR-252*-overexpressing wing discs exhibit inactivation of Notch signaling coupled with NICD accumulation due to the suppression of *Rab6*.

#### 4.3.3. *miR-31a/b* and *miR-8* in the Wg Signaling Pathway of Wing Development

The Wg pathway is one of the highly conserved signaling pathways, and the function of Wg signaling in the wing imaginal discs of *Drosophila* has been well elucidated [100]. In the wing discs, Wg is mainly expressed at the dorsal/ventral boundary [100]. Initially, Wg is synthesized within the Golgi apparatus and sorted into intracellular vesicles along with Wntless (Wls), a protein responsible for the secretion of Wg (Figure 4C). Subsequently, these Wg-Wls complexes are released into the extracellular space via exocytosis [101,102]. Upon release, the Wg ligand binds to the Frizzled (Fz) receptor, leading to the activation of Dishevelled (Dsh) in target cells. This inactivates the destruction complex, which comprises Adenomatous polyposis coli (APC), Axin (Axn), and Shaggy (Sgg). As a result, Armadillo (Arm) translocates into the nucleus and induces the expression of the Wg target genes, along with dTCF [103]. 

A conserved miRNA, *miR-31*, is implicated in the Wg signaling pathway by regulating transmembrane protein-encoding *wls* (Figure 4C), similar to its regulation in human cells [81,104]. Overexpression of both *miR-31a* and *miR-31b* in wing discs results in a significant reduction in the size of wing discs. Thus, miR-31 controls wing growth through the Wg signaling pathway. 

In addition, miR-8 inhibits Wg signaling by targeting *wls* in *Drosophila* wings (Figure 4C) [63]. Additionally, *miR-8* negatively regulates *CG32767* [63], which encodes a zinc-finger protein identified as a positive regulator of Wg signaling [105,106]. Upon *miR-8* overexpression along the anterior–posterior boundary of the wing pouch, the expression levels of *Wg*, Distal-less (*Dll*), and *Fz3* are substantially reduced [63]. 

### 4.4. miRNAs in Eye Development

*Drosophila* compound eyes consist of approximately 800 ommatidia [107]. Each ommatidium contains eight photoreceptors (R1–8), four cone cells, two primary pigment cells, six secondary pigment cells, three tertiary pigment cells, and three bristle complexes. Among the photoreceptors, the R1–6 cells are located in the outer regions, whereas the R7 and R8 cells are centrally located. The R7 cells are positioned above the R8 cells. 

**Figure 5:**
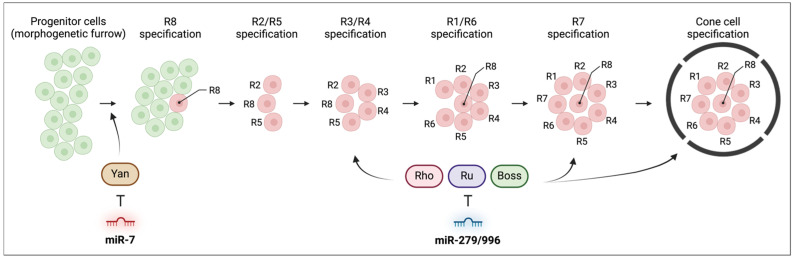
The roles of miRNAs in compound eye development of *Drosophila*. *miR-7* and *miR-279/996* regulate serial specification in the eye developmental process by modulating their target genes.

Eye development, including distinct cell fates and structural formation, is coordinated by multiple signaling pathways [107]. Following R8 photoreceptor specification mediated by Notch signaling, epidermal growth factor receptor (EGFR) and receptor tyrosine kinase (RTK) signaling facilitate the recruitment of R2/5, R3/4, R1/6, and R7 photoreceptors to each ommatidial cluster (Figure 5) [82,108]. Sevenless (Sev) receptor-mediated RTK signaling specifies the final photoreceptor, R7. 

*miR-7* is involved in photoreceptor differentiation in *Drosophila* eyes through the EGFR signaling pathway [80]. In situ hybridization confirmed that *miR-7* is expressed and active in the development of photoreceptors. In the ommatidia of *miR-7* deletion mutants, only ectopic R7 photoreceptors—and not ectopic cone cells—were observed, indicating that *miR-7* is specifically associated with photoreceptor differentiation. Additional analysis revealed that *miR-7* negatively regulates the E26 transformation-specific (ETS) domain transcription factor Yan (also known as anterior open [*aop*]), consequently stimulating photoreceptor differentiation (Figure 5) [80]. Furthermore, the derepression of Yan, mediated by the activation of EGFR signaling, induces the expression of *miR-7*, in addition to Yan regulation by *miR-7* [80]. This reciprocal regulation of *miR-7* and Yan controls eye development in *Drosophila*.

As for other eye development-associated miRNAs, *miR-279* and *miR-996* are essential for normal eye development [82]. *MiR-279* and *miR-996* are co-expressed and have identical seed sequences [109]. Deletion of *miR-279* and *miR-996* results in eye roughening [82]. When adult eyes are sectioned, seven of the eight photoreceptor rhabdomeres can be observed in the case of normal eyes, including six outer photoreceptors (R1–6) and one inner photoreceptor (either R7 or R8, depending on the position of the section). However, the adult eyes of *miR-279* and *miR-996* deletion mutants abnormally exhibit multiple ectopic R7 cells. Furthermore, in *miR-279* and *miR-996* mutants, the number of Arm + photoreceptor cells increases, while the number of cone cells decreases. These defects are associated with components of the RTK/EGFR signaling pathway, such as rhomboid (*rho*), roughoid (*ru*), and bride of sevenless (*boss*), which are regulated by *miR-279* and *miR-996* (Figure 5).

## 5. Discussion and Future Research Directions

In the last 20 years, numerous studies on *Drosophila* miRNAs have revealed a relationship between over 150 miRNA target genes. However, compared with the numerous potential target genes predicted for each miRNA by TargetScanFly, this number remains relatively small. Moreover, the biological roles of a substantial proportion of the 469 mature miRNAs in *Drosophila* remain unexplored. Thus, further investigation of miRNAs is essential for understanding gene regulation across diverse developmental processes. 

To comprehensively study the biological roles of miRNAs, it is critical to identify the target genes regulated by individual miRNAs. With the development and improvement of high-throughput sequencing techniques and computational biology tools, clearer information regarding the interactions between miRNAs and their target mRNAs can be obtained. CLIP-seq techniques, including high-throughput sequencing CLIP (HITS-CLIP), photoactivatable ribonucleoside-enhanced CLIP (PAR-CLIP), individual-nucleotide resolution CLIP (iCLIP), and enhanced CLIP (eCLIP), allow for the capture of RNAs that interact with specific RNA-binding proteins (Table 2) [110,111,112,113]. By applying this method to Ago proteins involved in miRNA pathways, Ago-binding miRNAs and Ago-binding mRNA regions can be identified. Then, using computational tools, substantial interactions between individual miRNAs and their target regions can be deduced. In *Drosophila*, several groups have identified these interactions using the Ago1-CLIP-seq method [50,51,114], facilitating an understanding of the regulatory network mediated by miRNAs. 

However, CLIP-seq is not a technique to directly capture miRNA–target mRNA interactions. To address this limitation, sequencing techniques, such as crosslinking, ligation, and sequencing of hybrids (CLASH) and covalent ligation of endogenous Argonaute-bound RNAs-CLIP (CLEAR-CLIP), have been developed and employed to study miRNA–target mRNA interactions in human cells (Table 2) [115,116]. Recently, RNA in situ conformation sequencing (RIC-seq), a method to capture global RNA-RNA interactions, has also been developed, allowing us to obtain information on miRNA–target mRNA interactions (Table 2) [117]. Therefore, to study the regulatory roles of individual miRNAs in *Drosophila* development, cutting-edge sequencing technologies are necessary. Due to the plasticity of the interactions between miRNAs and target mRNA, these techniques should be performed under various conditions, such as at different developmental stages and in different tissues. Through these approaches, the complex networks of miRNA–mRNA interactions will be revealed in various developmental processes. 

In addition, based on the vast interaction data obtained from sequencing techniques at each developmental time point and in specific tissues, comprehensive regulatory networks between multiple central miRNAs and their target genes should be simultaneously analyzed to understand complex developmental processes. However, to date, almost all studies have focused on only one or two miRNA target genes in a single context. In reality, one miRNA can regulate the expression of many genes by binding to specific regions within the 3′-UTR of multiple targets in an imperfect-match manner [118]. Conversely, multiple miRNAs can simultaneously modulate one target gene. Therefore, we should analyze the regulatory consequences mediated by miRNAs with different expression strengths and directions depending on the spatial and temporal conditions of development. 

Furthermore, compared with the expected miRNA–target gene interactions in *Drosophila* development, only a small portion of miRNA–target gene interactions have been experimentally validated. Numerous networks remain unknown under various developmental conditions. Therefore, in addition to sequencing methods, efforts are needed to uncover the individual relationships between miRNAs and target genes using various traditional approaches for miRNA studies, such as reporter assays. Through these efforts, it is expected that, in the near future, the puzzle of the regulatory mechanisms of miRNAs in developmental processes will be solved, leading to a broader understanding of the developmental processes of *Drosophila*. 

## Figures and Tables

**Table 1:** Summary of development-associated miRNAs and their target genes in Drosophila.

miRNA	Target Gene Name (Symbol)	Validation Methods ^1^	Tissues/Cells	References	Signaling Pathways
miR-252	mushroom bodies tiny (*mbt*)	Ago1 CLIP-seq, WB, Luc rep	Fat body	[58]	-
	Abelson interacting protein (*Abi*)	Ago1 CLIP-seq, WB, RT-qPCR, Luc rep	S2 cell	[50]	-
	Rab6 (*Rab6*)	Ago1 CLIP-seq, RT-qPCR, Luc rep	Wing	[59]	Notch
miR-276a	Insulin-like receptor (*InR*)	Ago1 CLIP-seq, Luc rep	Wing/fat body	[60]	IIS
	nejire (*nej*)	EGFP rep	Neuron	[61]	-
miR-8	u-shaped (*ush*)	RT-qPCR, WB, Luc rep	Fat body	[62]	IIS
	CG32767, wntless (*wls*)	Luc rep	Wing	[63]	Wg
miR-14	sugarbabe (*sug*)	Luc rep, EGFP rep	Insulin-producing neurosecretory cell	[64]	IIS
	Death related ICE-like caspase (*Drice*)	WB	Eye	[65]	Apoptosis
Bantam	head involution defective (*hid*)	EGFP rep, IS	Wing	[66]	Apoptosis
	brain tumor (*brat*), prospero (*pros*)	RT-qPCR, Luc rep	Neuroblast	[67]	-
miR-6	head involution defective (*hid*), reaper (*rpr*), grim (*grim*), sickle (*skl*)	RT-qPCR, Luc rep	Central nervous system	[68]	Apoptosis
miR-11					
let-7	Chronologically inappropriate morphogenesis (*chinmo*)	Luc rep, IS	Mushroom body	[69]	-
	abrupt (*ab*)	IS	Mushroom body	[70]	-
miR-iab8	CG12229, Ceramide phosphoethanolamine synthase (*Cpes*)	Behavioral analyses	Mushroom body	[71]	-
miR-92a/b	Jing interacting gene regulatory 1 (*jigr1*)	RT-qPCR, IS, WB, Luc rep	Neuroblast	[72]	-
miR-124	anachronism (*ana*)	RT-qPCR, Luc rep	Neuroblast	[73]	-
	saxophone (*sax*), wishful thinking (*wit*), thickveins (*tkv*), Mothers against dpp (*Mad*), Medea (*Med*)	Luc rep	Central nervous system	[74]	-
miR-34	Neurexin IV (*Nrx-IV*), hu li tai shao (*hts*)	IS	Central nervous system	[75]	-
miR-960	smoothened (*smo*), costa (*cos2*), fused (*fu*)	Luc rep, IS, EGFP rep	Wing	[76]	Hh
miR-5	smoothened (*smo*)	Luc rep, EGFP rep, IS	Wing	[77]	Hh
miR-7	Enhancer of split complex (*E*(*spl*)*-C*)	EGFP rep	Wing	[78,79]	Notch
	*Yan* (=anterior open (*aop*))	EGFP rep, IS	Eye	[80]	-
miR-31a/b	wntless (*wls*)	RT-qPCR, WB	Wing	[81]	Wg
miR-279/996	rhomboid (*rho*), roughoid (*ru*), bride of sevenless (*boss*)	EGFP rep, Luc rep	Eye	[82]	-

^1^ WB, Western blotting; Luc rep, luciferase reporter assay; EGFP rep, EGFP reporter assay; IS, immunostaining.

**Table 2:** Sequencing techniques for identification of miRNA targets.

Sequencing Techniques		Features ^1^	Reference
RNA–Protein interaction	HITS-CLIP	- High-throughput CLIP-seq method using 254 nm UVC - Identifies mRNA fragments and miRNAs associated with Ago protein - Requires additional bioinformatic analysis to confirm miRNA–mRNA interactions	[110]
	PAR-CLIP	- Modified from HITS-CLIP - Uses 4-thiouridine and 312–365 nm UVA/B to enhance RNA–protein crosslinking	[111]
	iCLIP	- Modified from HITS-CLIP - Improves efficiency using DNA circularization after reverse transcription	[112]
	eCLIP	- Modified from HITS-CLIP - Enhances efficiency using adapter ligation after reverse transcription	[113]
RNA–RNA interaction	CLASH	- Direct ligation of miRNA and mRNA fragments - Includes Ago protein denaturation and two purification steps	[115]
	CLEAR-CLIP	- Direct ligation of miRNA and mRNA fragments - Single purification step; does not require full denaturation of Ago	[116]
	RIC-seq	- Captures global RNA–RNA interactions using in situ proximity ligation with pCp-biotin - May require Ago immunoprecipitation to enrich miRNA–mRNA interactions	[117]

^1^ It described only the basic characteristics and features of the method from the perspective of identifying miRNA targets.

## Data Availability

Data are contained within the article.
